# Efficacy of Drug-Eluting Stents in Diabetic Patients Admitted with ST-Elevation Myocardial Infarctions Treated with Primary Percutaneous Coronary Intervention

**DOI:** 10.3390/jcdd8080083

**Published:** 2021-07-21

**Authors:** Johannes Schmucker, Andreas Fach, Rico Osteresch, Luis Alberto Mata Marin, Stephan Ruehle, Tina Retzlaff, Daniela Garstka, Ingo Eitel, Rainer Hambrecht, Harm Wienbergen

**Affiliations:** 1 Bremen Institute for Heart and Circulation Research, 28277 Bremen, Germany; andreas.fach@klinikum-bremen-ldw.de (A.F.); rico.osteresch@klinikum-bremen-ldw.de (R.O.); luisalberto.matamarin@klinikum-bremen-ldw.de (L.A.M.M.); Stephan.Ruehle@gesundheitnord.de (S.R.); Tina.Retzlaff@klinikum-bremen-ldw.de (T.R.); Daniela.Garstka@klinikum-bremen-ldw.de (D.G.); rainer.hambrecht@klinikum-bremen-ldw.de (R.H.); Harm.wienbergen@klinikum-bremen-ldw.de (H.W.); 2 Medical Clinic II, University Heart Center, 23562 Lübeck, Germany; Ingo.eitel@uksh.de

**Keywords:** STEMI, diabetes mellitus, drug-eluting stents, coronary artery disease

## Abstract

Background: Diabetic patients show higher adverse ischemic event rates and mortality when undergoing percutaneous coronary intervention (PCI) in acute myocardial infarctions. Therefore, diabetic patients might benefit even more from modern-generation drug-eluting stents (DES). The aim of the present study was to compare adverse ischemic events and mortality rates between bare-metal stents (BMS) and DES in diabetic patients admitted with ST-elevation-myocardial infarction (STEMI) with non-diabetic patients as the control group. Methods: All STEMI patients undergoing emergency PCI and stent implantation documented between 2006 and 2019 in the Bremen STEMI registry entered the analysis. Efficacy was defined as a combination of in-stent thrombosis, myocardial re-infarction or additional target lesion revascularization at one year. Results: Of 8356 patients which entered analysis, 1554 (19%) were diabetics, while 6802 (81%) were not. 879 (57%) of the diabetics received a DES. In a multivariate model, DES implantation in diabetics compared to BMS was associated with lower rates of in-stent thrombosis (OR 0.16, 95% CI 0.05–0.6), myocardial re-infarctions (OR 0.35, 95%CI, 0.2–0.7, *p* < 0.01) and of the combined endpoint at 1 year ((ST + MI + TLR): OR 0.31, 95% CI 0.2–0.6, *p* < 0.01), with a trend towards lower 5-year mortality (OR 0.56, 95% CI 0.3–1.0, *p* = 0.058). When comparing diabetic to non-diabetic patients, an elevation in event rates for diabetics was only detectable in BMS (OR 1.78, 95% CI 0.5–0.7, *p* < 0.01); however, this did not persist when treated with a DES (OR 1.03 95% CI 0.7–1.6, *p* = 0.9). Conclusions: In STEMI patients with diabetes, the use of DES significantly reduced ischemic event rates and, unlike with BMS, adverse ischemic event rates became similar to non-diabetic patients.

## 1. Introduction

Patients with diabetes mellitus are at an increased risk of developing coronary artery disease (CAD) [1,2]. Furthermore, recent years have shown a rise in diabetes prevalence worldwide [3]; thus, the importance of diabetes in CAD is likely to increase. Previous studies have consistently shown that diabetes mellitus is associated with a poorer outcome after percutaneous coronary intervention (PCI), with higher rates of re-stenosis occurring, as well as higher incidences of death and myocardial infarctions [4,5]. Since diabetes mellitus is associated with a higher likelihood of complex CAD [6] the choice of intervention is crucial in determining long-term outcome. The FREEDOM trial has shown that coronary-artery bypass grafting (CABG) was superior to percutaneous coronary intervention (PCI), resulting in reduced rates of death and myocardial infarction, with an increased risk of stroke [7]. However, patients with recent acute ST-elevation myocardial infarctions (STEMI) were excluded from the FREEDOM trial and recent data from the Netherlands has shown that only a minority of patients with STEMI undergo emergency CABG, which limits the therapeutic options for STEMI patients with diabetes even in the presence of advanced CAD [8]. Over the last 10 to 15 years, modern drug-eluting stents (DES) have been increasingly used in patients with STEMIs and are recommended in current guidelines from the European Society of Cardiology (ESC) [9]. DES have shown to be superior to bare-metal stents (BMS), leading to lower target-lesion or target-vessel-revascularization (TLR/TVR) rates [10] and, in some studies, lower rates of myocardial reinfarctions [11]. However, data on the efficacy of drug-eluting stents in patients with diabetes and complex CAD and acute myocardial infarctions is scarce.

The hypotheses of this study were that, firstly, ischemic event rates were to be higher for diabetic STEMI patients, and, secondly, that modern DES should lower adverse ischemic event rates especially in diabetics, which could lead to an attenuation of their disadvantage compared to non-diabetics. 

## 2. Methods

### 2.1. The Bremen STEMI Registry (BSR)

STEMI patients from the metropolitan area of Bremen in Northwest Germany (~1,000,000 inhabitants) which are admitted at the Bremen Heart center, have, since 2006, been documented in the Bremen STEMI Registry (BSR). Emergency services and regional hospitals are closely connected by telephone and fax with the interventional center for rapid communication to enable urgent percutaneous coronary interventions (PCI). Documentation for the BSR is done via data sheets completed by the responsible interventional cardiologist and/or through patient records after a cardiologist has confirmed the diagnosis. Data about age, sex, concomitant diseases, severity of STEMI, acute medical or interventional treatment, as well as laboratory parameters at admission and during the hospital stay are recorded. After discharge, major adverse cardiac and cerebral events as well as bleeding events are documented through follow-up examination performed after 1, 5 and 10 years by a telephone interview. Before inclusion of patient records in the database, a written consent on study participation was obtained. The study was approved by the ethical committee of the Ärztekammer Bremen, Germany. Studies about methods and results from the BSR have been previously published elsewhere [12,13,14,15,16,17,18]. 

For this study all patients which did undergo coronary catheterization but did not undergo PCI and stent-implantation were excluded. 

### 2.2. Definition of STEMI 

STEMI was defined as persistent angina pectoris for ≥20 min in conjunction with a ST-segment elevation in two contiguous leads of ≥0.25 mV in men below the age of 40 years, ≥0.2 mV in men over the age of 40 years, or ≥0.15 mV in women in leads V2–V3 and/or ≥0.1 mV in all other leads or new left bundle branch block (LBBB) [9].

Subacute STEMIs were defined as STEMIs with >12 h between first symptoms and first medical contact and/or signs of a subacute myocardial infarction in the ECG at admission.

### 2.3. Definition of Outcomes

In-hospital outcomes were evaluated at discharge or at time of patient-transfer to a local hospital. 30 day, 1-year- and 5-year-follow-up outcomes were evaluated in a telephone interview. To estimate efficacy, the primary endpoint was defined as a combination of in-stent thrombosis, myocardial reinfarction and repeat target lesion revascularizations (TLR) within 1 year. Overall mortality was assessed at 1 and 5 years. The safety endpoint was defined as a combination of in-hospital bleedings (TIMI minor and major) or any significant bleeding event after hospital discharge within 1 year. 

### 2.4. Statistical Analysis

All patients admitted with STEMI to the Bremen Heart center between 1 January 2006 and 30 June 2019 were initially assessed. For further analysis, all patients which were not treated with primary PCI or without a stent-implantation were excluded. For calculation of 5-year mortality rates, only patients admitted between 2006 and March and 2015 were analysed. Baseline characteristics of patients were described by mean values and standard deviations (SD) or standard error of mean (SEM) for continuous variables. Absolute numbers and percentages were reported for categorical variables. Univariate comparison was done with Mann–Whitney U tests for continuous variables (since no normal distribution was found) and chi-square tests for categorical variables. For the multivariate comparison, a logistic regression analysis was used. To analyse the impact of stent type in diabetic patients, adverse and safety events were defined as the dependent variable, while stent type, age, gender, cardiogenic shock (CS), PCI result, number of stents, type of P2Y12 inhibitor and duration and location of STEMI were defined as the independent covariates. For multivariate comparison of the impact on diabetes status on outcome, a separate model was calculated for BMS- and DES-treated patients, with adverse events as the dependent variables and diabetic status, age, gender, CS, PCI result, number of stents, type of P2Y12 inhibitor and duration and location of STEMI as the independent covariates. The choice of variables was made a priori on the basis of consensual clinical judgement and established cardiovascular risk factors. 

All calculations were done with SAS, from SAS-Institute, Inc. (Cary, NC, USA) 2018. 

## 3. Results

### 3.1. Study Population 

**Figure 1:**
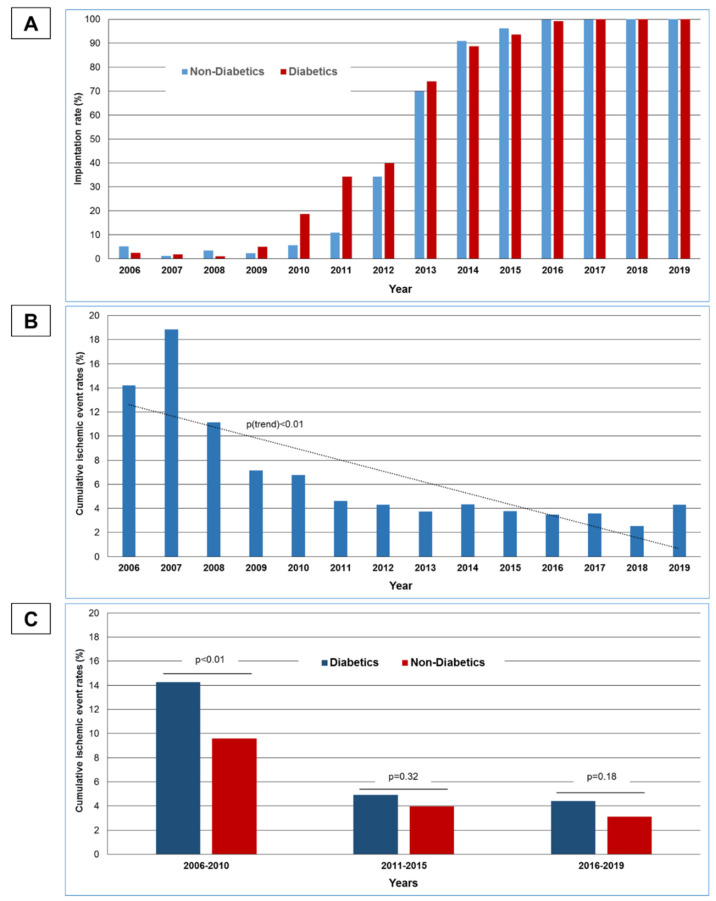
Trends in stent types and adverse ischemic events: (**A**) Proportion of STEMI patients treated with DES in diabetics and non-diabetics 2006–2019. (**B**) Development of cumulative ischemic event rates (ST + AMI + TLR) 2006–2019 in diabetics and non-diabetics. (**C**) Comparison of cumulative ischemic event rates (ST + AMI + TLR) in diabetic and non-diabetic patients in the study cohort.

Between 1 January 2006 and 30 June 2019, 9890 patients were admitted with STEMI to the Bremen Heart Center and documented in the Bremen STEMI Registry. For further analysis, 992 patients were excluded because they were not treated with a primary PCI, and 542 patients were excluded, because no stent was implanted during PCI. Of the remaining 8356 patients, 6802 (81%) were non-diabetic patients, while 1554 (19%) had diabetes mellitus. Of the non-diabetic patients, 3210 (47%) were treated with a BMS, while 3592 (53%) received a DES. Of the diabetic STEMI patients, 675 (43%) were treated with a BMS, and 879 (57%) with a DES. Over time (2006–2012 vs. 2013–2019) no significant change in mean age of the diabetic STEMI patients could be seen (66.6 ± 12 to 66.9 ± 12 yrs., n.s.), nor could any change be seen in the proportion of women (34.5% to 33.9%, n.s.). There was, however, a rise in diabetic STEMI patients presenting with cardiogenic shock (9.6% to 16.3%, p < 0.01) and with multivessel-disease (68.8% to 73.3%, p = 0.05). During the study period, an increase in the deployment rate of DES could be seen in diabetic as well as in non-diabetic STEMI patients (Figure 1A).

### 3.2. Comparison of Diabetic vs. Non-Diabetic Patients with STEMI

Diabetic patients were on average 4.2 years older than non-diabetic patients, more likely to be female and more likely to be obese; however, diabetic patients showed lower rates of active smoking. Diabetic STEMI patients were more likely to have known CAD, cerebrovascular disease or peripheral artery disease compared to non-diabetic patients. Furthermore, they were more likely to have multi-vessel disease at the index event and more likely to present with a subacute STEMI, while rates of STEMI complicated by cardiogenic shock (CS) were similar. Interventional success rates were similar between diabetic vs. non-diabetic patients, and while diabetic patients showed on average smaller infarctions, they conversely presented with higher rates of an at least moderately decreased LVEF after STEMI (Table 1). Diabetic patients were more likely to be treated with a dual antiplatelet therapy (DAPT) with clopidogrel; however, differences were small (43.9% vs. 40.4%). Diabetics were more likely to receive ticagrelor, while treatment rates with prasugrel were higher for non-diabetic patients. Diabetics were less likely to be treated with an ACE-inhibitor/angiotensin II receptor blocker (ARB), while treatment rates with a mineralocorticoid receptor antagonist (MRA) or a beta-blocker were similar. Of the diabetics, in nearly one third only a dietary regime was sufficient, in 34.3% at least one oral antidiabetic medication was given at discharge and in another 37.7% a therapy including insulin was necessary (Table 1).

While over time, 1-year event rates of the combined efficacy endpoint (ST + AMI + TLR) showed a significant decrease in the combined STEMI population (Figure 1B), this decrease was more pronounced in diabetics than in non-diabetics over time (Figure 1C). While during the first years of the study period diabetics showed significantly higher rates of the primary efficacy endpoint, this disadvantage for diabetics was no longer significant during the last study years (Figure 1C).

### 3.3. Diabetic Patients with DES vs. BMS: Baseline Characteristics, Interventional Details and Medication at Discharge

When focusing on diabetic patients and comparing stent types, DES patients were on average 2 years younger, while other baseline characteristics were similar. Patients treated with a DES were more likely to have known CAD before the index event and were more likely to present with cardiogenic shock (CS) and with complex CAD at the index event, while rates of unsuccessful PCI were similar between stent groups. Neither size of STEMI, estimated by peak-CK, nor LVEF post-STEMI differed between stent groups. However, the use of DES in diabetic patients was associated with, on average, a higher number of stents implanted (Table 2). Due to changes in treatment strategies, patients after DES implantation were more likely to be treated with the modern P2Y12-inhibitors ticagrelor and prasugrel; however, they were less likely to be treated with clopidogrel or a triple therapy. Furthermore, duration of treatment with a dual antiplatelet therapy (DAPT) was on average more than 7 months longer for patients who had received a DES compared to BMS. DES patients were less likely to be treated with a beta-blocker and more likely to be treated with an MRA post-STEMI (Table 2).

### 3.4. Diabetic Patients with DES vs. BMS: In-Hospital Event Rates and Outcome after 1 Year

During the hospital stay, patients treated with a DES had significantly higher in-hospital mortality rates, while rates of in-hospital resuscitations and in-hospital strokes were similar. Furthermore, while minimal bleeding events were more frequent in patients treated with DES, there were no differences in rates of TIMI minor or major bleedings during the hospital stay. Patients treated with a DES were more likely to undergo a staged procedure, meaning that on at least one occasion a planned repeat PCI on another vessel was performed within one year after the index event. However, staged procedure rates in DES and BMS were comparatively low (Table 3). 

At 1 year, patients treated with DES showed significantly lower rates of stent thrombosis, myocardial re-infarction and repeat target lesion revascularisation. The combined primary efficacy endpoint (ST + AMI + TLR) therefore occurred more than 2.5 times more often in BMS than in DES (12.2% vs. 4.2%, *p* < 0.01). 

In diabetic patients, bleeding events at 1 year were higher with DES, while all-cause mortality at 1 year did not differ between stent types in diabetics. There was, however, a significant reduction in 5-year mortality in DES-treated diabetic patients (Table 3).

**Figure 2:**
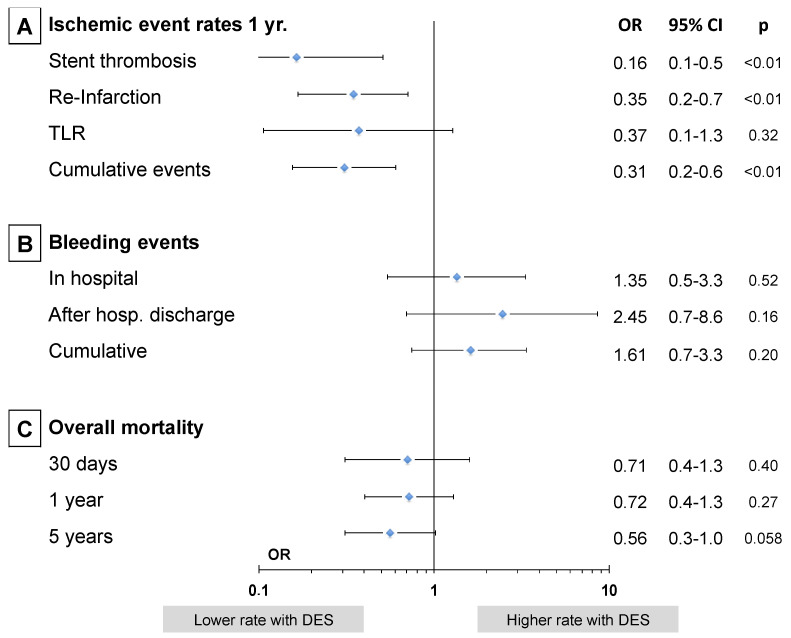
Event rates in diabetic STEMI patients treated with DES vs. BMS: Comparison of (**A**) ischemic events, (**B**) bleeding events and (**C**) all-cause mortality. Multivariate model with stent type adjusted for age, gender, cardiogenic shock, anterior STEMI, type and duration of P2Y12-inhibitor and number of stents implanted. Cumulative events = ST + AMI + TLR.

The results of the univariate comparisons in diabetics could largely be confirmed in a multivariate model: The use of DES was associated with significantly lower adverse ischemic event rates (Figure 2A). To assure that this advantage was not triggered by higher rates of planned staged PCIs in DES patients (Table 3), event rates were furthermore adjusted for any staged-PCI events within 1 year. Despite this, the advantage for DES remained significant: OR 0.337, 95% CI 0.17–0.66, *p* < 0.01. Furthermore, when adjusting the multivariate model for the use of beta-blockers and MRAs, the advantage for DES with regard to ischemic event rates remained significant: 0R 0.42, 95% CI 0.24–0.73, *p* < 0.01.

When analysing safety events, unlike in the univariate comparison, a multivariate model showed that bleeding rates did not differ significantly between DES and BMS cohorts (Figure 2B). Furthermore, 30-day and 1-year mortality were not significantly reduced in DES-treated patients; however, there was a strong trend towards lower 5-year mortality rates in DES-treated patients (Figure 2C). 

**Figure 3:**
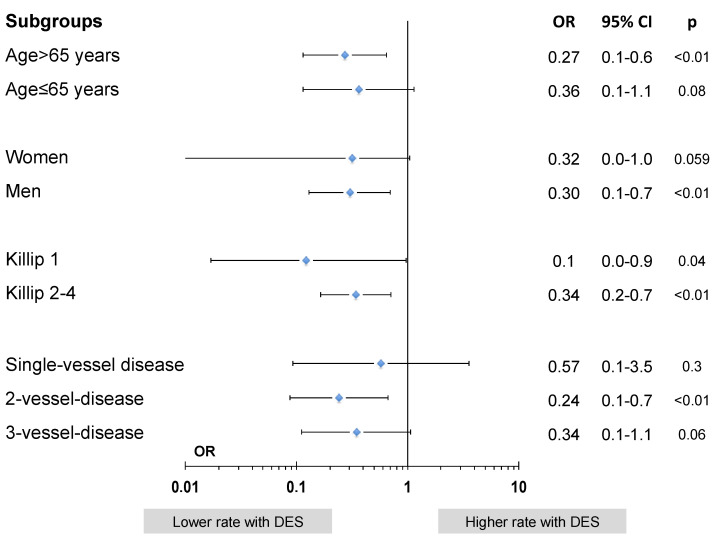
Subgroup analysis of cumulative ischemic event rates in DES vs. BMS: Multivariate model analysis for subgroups with stent type adjusted for age, gender, cardiogenic shock, anterior STEMI, type and duration of P2Y12-inhibitor and number of stents implanted.

In a subgroup analysis of the primary efficacy endpoint, it was shown that in all subgroups (divided by age, gender, CS or severity of CAD) a numerically comparable reduction of the combined ischemic event rates could be observed, while in specific groups this failed to reach statistical significance (Figure 3).

### 3.5. Univariate and Multivariate Analysis of Efficacy and Safety of DES vs. BMS in Diabetic Compared to Non-Diabetic Patients 

When comparing diabetic vs. non-diabetic patients and stratifying patient-cohorts by stent-type, a univariate comparison showed that while cumulative ischemic event rates were higher for diabetic patients treated with a BMS (12.2 vs. 7.5%, *p* < 0.01), this disadvantage was no longer evident in DES (4.2 vs. 3.7%, *p* = 0.49, Table 4). 

**Figure 4:**
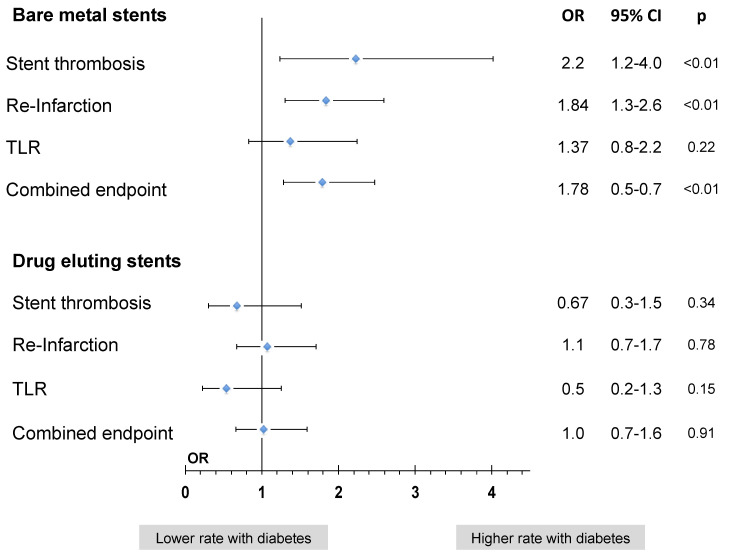
Impact of diabetes on ischemic event rates stratified by stent type: Comparison of adverse ischemic event rates in diabetic vs. non-diabetic patients in patients treated with BMS and DES. Multivariate model with diabetes adjusted for age, gender, cardiogenic shock, anterior STEMI, type and duration of P2Y12-inhibitor and number of stents implanted. Combined endpoint = ST + AMI + TLR.

A multivariate analysis confirmed these findings. While in BMS-treated cohorts, diabetes was associated with a significantly higher likelihood of the combined ischemic endpoint within 1 year after the index event, this disadvantage was no more evident in DES-treated cohorts (Figure 4).

When analysing each of the components of the combined endpoint in the univariate and multivariate model, both stent thrombosis and myocardial re-infarctions were significantly more likely to occur in diabetic patients when treated with a BMS; again, this disadvantage for diabetics was no longer evident for diabetics when treated with a DES (Figure 4).

Bleeding rates did not differ between diabetic and nondiabetic patients regardless of stent type; however, bleeding rates in DES were generally higher. While 1-year-overall-mortality rates were higher for diabetic compared to non-diabetic patients regardless of stent-type, 5-year overall mortality was only elevated for diabetic patients when treated with a BMS, not in patients treated with a DES (Table 4).

## 4. Discussion

The present study shows that in diabetic patients undergoing emergency PCI, DES compared to BMS significantly reduced ischemic events and, as a trend, lowered 5-year mortality rates, even in the presence of multi-vessel disease. 

While when BMS were widely in use, diabetic patients were at a disadvantage compared to non-diabetics, showing higher adverse ischemic event rates, this difference was no longer evident when DES were predominantly implanted. Overall mortality, however, remained higher for diabetic STEMI patients, even when DES were used.

### 4.1. Impact of Diabetes on Adverse Ischemic Events and Mortality in Patients with CAD and CAD-Events

Diabetes is a known cause of CAD and acute myocardial infarctions. Haffner et al. showed that diabetics without known CAD where at a similar risk of suffering an AMI as patients with known CAD and no diabetes [2]. Furthermore, diabetes is also associated with worse outcomes, both after elective PCI and after acute coronary syndromes. A pooled analysis of 11 TIMI trials, published in 2007 by Donahoe et al., demonstrated that in an adjusted analysis, diabetes was associated with a 65% elevation in mortality at one year in patients with unstable angina/NSTEMI and a 22% elevation in mortality after STEMI [19]. This disadvantage was evident both in patients undergoing revascularisation procedures and those who were conservatively treated. A recent analysis of PCI patients from New York confirmed these findings in more actual data: At one year, diabetes was associated with a 2.1× elevation in adverse event rates (MACE) in insulin-treated DM (ITDM) and with a HR of 1.27 in non-ITDM after PCI [20]. In the GRACE multinational registry, diabetes proved to be an independent factor to predict in-hospital and 6-month mortality in patients with ACS. While diabetes did not meet the criteria to be included in the GRACE risk prediction tool, this was probably mainly due to the necessity to simplify the score calculation by limiting the number of variables [21]. Diabetes, however, was included in other risk prediction tools like the TIMI risk scores for STEMI and UA/NSTEMI [22,23]. 

There are multiple reasons for the negative impact of diabetes on long-term outcomes in CAD patients. First, diabetic patients are more likely to have complex CAD^6^. In an autopsy cohort study from Olmsted County, Minnesota, diabetics were more likely to have advanced atherosclerosis than non-diabetics, with a high proportion of patients showing multi-vessel disease, even when they had no known CAD [24]. Second, diabetic CAD patients are more likely to have more comorbidities, often due to the detrimental effects on organs like diabetic nephropathy and diabetic neuropathy, which partly explains why diabetics are more likely to be treated >6 h after symptom onset in AMI [25]. Third, in diabetic patients with CAD, a diabetic cardiomyopathy (DC) is often described, which might contribute to a worse overall prognosis, although it remains unclear whether DC is truly independent of an ischemic origin [26]. 

In the present study all the prior descriptions of diabetes in CAD could be reproduced: Diabetic STEMI patients in this study cohort had more cardiovascular comorbidities, a higher likelihood of complex CAD at the index event, a higher likelihood of a reduced left ventricular function after STEMI and a worse outcome at one year compared to non-diabetic STEMI patients.

### 4.2. Choice of Revascularization Strategies and Stent Type in Diabetic Patients with Complex CAD

Randomized trials comparing revascularisation strategies in diabetic patients with coronary multi-vessel disease (MVD) have mostly found that CABG was superior to PCI with regard to long-term outcome. The BEST trial, which enrolled patients between 2008 and 2013, showed that in diabetic patients with MVD, CABG was superior to PCI with everolimus-eluting stents, demonstrating lower rates of the combined primary endpoint of death, myocardial infarction or target vessel revascularization (TVR) in the CABG group [27]. However, this difference was mostly triggered by higher rates of a repeat TVR in the PCI group [27]. The FREEDOM trial, which enrolled patients between 2005 and 2010, confirmed that in diabetic patients with MVD, CABG was superior to a PCI with a DES. Unlike in the BEST trial, this superiority was not caused by lower rates of repeat revascularizations, but also by lower rates of myocardial infarction and all-cause mortality at five years [7]. This survival benefit for CABG could be confirmed in a long-term follow-up at eight years [28]. However, both trials enrolled their patients when mostly older-generation stents were still in use, with supposedly higher adverse ischemic events rates after PCI, as could be seen in our data during the first years of the study period. Second, the benefit for CABG was triggered at least partially by repeat target vessel or target lesion revascularisations, which were less likely to occur in our registry data with newer-generation stents. Third, both trials excluded patients with acute myocardial infarction, which comprised the cohort in the present study. In STEMI, the interventional cardiologist is often left with no other option, even in patients with diabetes and MVD, since emergency CABG is only recommended if PCI is not feasible or a PCI attempt failed [9].

The COMFORTABLE-AMI trial compared the use of DES and BMS in STEMI patients in a randomized trial, showing that the use of a DES was associated with lower rates of the combined endpoint of cardiovascular death, target vessel reinfarction, or ischemia-driven target lesion revascularization at 1, 2 and 5 years [11,29,30]. This benefit was only evident in non-diabetic patients at one year, while diabetic patients did not benefit from the use of DES [29]. This differs from the findings in the present study; however, the diabetic study cohort in the COMFORTABLE-AMI trial were considerably smaller, with 84 in the BMS group compared to 675 in the present study, and 90 in the DES group compared to 974 in our analysis. A comparison of BMS and DES especially in diabetic patients has been summarised in a recent meta-analysis by Bundhun et al. [31]. Use of DES was only associated with a reduction in TLR and TVR, while no difference in overall mortality or myocardial infarction could be detected. Previous findings on the efficacy of DES in diabetic vs. non-diabetic patients with ACS have been inconsistent: While a study by Lee [32] et al. reported that in ACS patients treated with a DES, diabetics continued to have an excess risk of death and major adverse cardiac events at one year, a study by Syed et al. with a similar cohort failed to find a disadvantage for diabetics, when adjusting event rates for other co-morbidities [33]. However, both studies analysed data at a time when DES were not routinely implanted in ACS patients. A study from the German ALKK-PCI registry showed that in STEMI patients treated between 2006 and 2011, a DES was only implanted in 21.5% of the total cohort and in 31.7% of patients with preexisting diabetes [34]. These rates are consistent with the implantation rates in the present study.

To our knowledge our study is the first investigation focusing on the possible benefit of DES in diabetic patients with STEMI, showing that DES greatly reduced ischemic events within one year after the event with now-comparable rates compared to non-diabetic patients.

### 4.3. Concurrent Changes in P2Y12-Inhibitors and Duration of DAPT

Besides the increasing use of DES, the other major change in STEMI therapy during the study period was the introduction of the more potent P2Y12-inhibitors ticagrelor and prasugrel as standard treatment after PCI, and the prolongation of the recommended duration of a dual antiplatelet therapy (DAPT). 

This might have influenced a direct comparison of the diabetic BMS and DES-treated cohorts. Bleeding events after hospital discharge occurred more often after DES-implantation; however, this effect should at least be partially contributed to the prolonged and more potent P2Y12 inhibition. 

Despite adjusting the model in a multivariate analysis, residual confounding should still be expected. However, the direct comparison of diabetics and non-diabetics, stratified by stent type, should not be influenced in the same way by other changes in therapy when a similar efficacy of a more potent P2Y12 inhibition or a longer duration of DAPT in both diabetics and non-diabetics is assumed. 

A sub-study from the PLATO study did not show any differences of efficacy of ticagrelor in diabetic versus non-diabetic patients, though event rates for diabetic patients were generally higher [35]. A sub-study from the TRITON-TIMI-38 trial, in contrast, observed a greater clinical benefit from prasugrel in diabetics compared to non-diabetics with regard to the primary endpoint; however, since this sub-study was not sufficiently powered, this could only be seen as an exploratory finding [36]. Furthermore, only 26% of the patients in the TRITON-TIMI-38 trial had the diagnosis of STEMI, so comparability to the present study is limited.

## 5. Limitations

No details on severity of diabetes or current therapy, like HbA1c-levels, prior duration of DM, type of diabetes (type 1 or 2) or specifics of medical therapy could be provided since they were not documented in this registry. Furthermore, this study is limited, since, although in the multivariate models treatment groups were adjusted for confounders, residual confounding is to be expected. The current registry does not include specific information on the exact stent type. We therefore could not compare the possible benefit of newer-generation DES to older-generation DES or BMS with regard to the primary efficacy outcome.

## 6. Conclusions

This study demonstrates that in diabetic STEMI patients undergoing emergency PCI, the use of DES reduced ischemic events and, as a trend, lowered 5-year mortality rates. When treated with a BMS, diabetic patients were still at a significant disadvantage with higher adverse ischemic events compared to non-diabetics; however, this was no longer evident when treated with a DES. Overall mortality rates, however, remained higher for diabetic STEMI patients, irrespective of type of stent used.

## Figures and Tables

**Table 1:** Characteristics of diabetic vs. non-diabetic patients with STEMI.

	Diabetics (n = 1554)	Non-Diabetics (n = 6802)	*p* Value
Age (years ± SD)	66.7 ± 12.1	62.5 ± 13.3	<0.01
Women (%)	34.1	25.5	<0.01
Body mass index (BMI) (kg/m² ± SD)	29.5 ± 5.5	27.2 ± 4.5	<0.01
Obesity (BMI ≥ 30 kg/m²) (%)	40.2	21.6	<0.01
Current smokers (%)	29.4	47.0	<0.01
**Medical history**			
Coronary artery disease (%)	22.7	13.3	<0.01
PCI (%)	15.8	10.3	<0.01
Acute myocardial infarction(%)	16.9	10.3	<0.01
CABG (%)	4.2	1.7	<0.01
Stroke/TIA (%)	8.7	4.4	<0.01
Peripheral artery disease (%)	6.8	4.0	<0.01
Subacute STEMI (%)	13.3	10.7	<0.01
Anterior STEMI or new LBBB (%)	47.3	49.8	0.05
Cardiogenic shock (%)	13.1	13.3	0.9
**Coronary vessels diseased**			
1 (%)	28.8	41.7	<0.01
2 (%)	34.7	31.9	0.036
3 (%)	36.5	26.4	<0.01
No flow/low reflow after PCI (%)	2.3	1.8	0.18
**Stent type**			
Bare-metal stent (%)	43.4	47.2	<0.01
Drug-eluting stent (%)	56.6	52.8	<0.01
Peak creatine kinase (mean ± SEM)	1738 ± 10	2029 ± 28	<0.01
LVEF < 40% post-STEMI (%)	17.1	12.2	<0.01
**Concomitant medication at discharge**			
ASS (%)	97.0	97.2	0.17
Clopidogrel (%)	43.9	40.4	0.012
Ticagrelor (%)	18.3	15.5	<0.01
Prasugrel (%)	35.0	42.3	<0.01
ACE-inhibitior/ARB (%)	80.6	83.6	0.01
Betablocker (%)	76.5	76.7	0.8
MRA (%)	15.6	16.4	0.69
Diabetic therapy: diet only	32.1	-	-
Diabetic therapy: oral antidiabetic	34.3	-	-
Diabetic therapy: insulin and/or OAD	33.7	-	-

**Table 2:** Characteristics of diabetic patients with STEMI treated with BMS vs. DES.

	BMS (n = 675)	DES (n = 879)	*p* Value
Age (years ± SD)	67.9 ± 11.8	65.9 ± 12.3	<0.01
Women (%)	35.1	33.3	0.31
Body mass index (BMI) (kg/m²)	29.6 ± 5.4	29.5 ± 5.6	0.9
Obesity (BMI > 30 kg/m²) (%)	40.4	40.0	0.5
Current smokers (%)	30.9	28.2	0.18
**Medical history**			
Coronary artery disease (%)	19.4	24.6	0.02
PCI (%)	11.6	18.4	<0.01
Acute myocardial infarction(%)	14.9	18.0	0.12
CABG (%)	4.4	4.1	0.53
Stroke/TIA (%)	8.5	8.9	0.66
Peripheral artery disease (%)	7.1	6.5	0.66
**Clinical presentation**			
Subacute STEMI (%)	13.0	13.5	0.63
Anterior STEMI or new LBBB (%)	43.6	50.2	0.02
Initial syst. BP (mmHg ± SD)	131.4 ± 29.5	130.7/±28.9	0.37
Cardiogenic shock (%)	10.1	15.4	<0.01
**Coronary vessels diseased**			
1 (%)	31.9	26.5	0.019
2 (%)	36.6	33.2	0.17
3 (%)	31.4	40.3	<0.01
No reflow after PCI (TIMI 0/1) (%)	2.8	1.8	0.19
Peak creatine kinase (mean ± SEM)	1749 ± 71	1723 ± 68	0.96
Mean number of stents implanted (n ± SD)	1.29 ± 0.6	1.54 ± 0.8	<0.01
>2 stents implanted (%)	5.0	11.8	<0.01
LVEF < 40% post-STEMI	18.1	16.6	0.53
**Concomittant medication at discharge**			
ASS (%)	98.8	95.5	<0.01
Clopidogrel (%)	76.8	18.7	<0.01
Ticagrelor (%)	5.3	28.3	<0.01
Prasugrel (%)	15.6	49.9	<0.01
Triple Therapy (%)	19.5	8.2	<0.01
Duration of DAPT (months ± SD)	4.1 ± 3.6	11.4 ± 2.2	<0.01
ACE-inhibitor/ARB (%)	79.1	81.7	0.26
Betablocker (%)	82.2	72.5	<0.01
MRA (%)	10.8	19.3	<0.01

**Table 3:** Outcome of diabetic patients with STEMI treated with BMS vs. DES.

	BMS (n = 675)	DES (n = 879)	*p* Value
In-hospital mortality (%)	6.9	10.1	0.028
In-hospital resuscitations (%)	3.3	4.8	0.14
In-hospital strokes	0.5	0.5	0.9
**In-hospital bleeding events**			
TIMI minimal (%)	5.6	10.5	<0.01
TIMI minor (%)	2.4	1.6	0.3
TIMI major (%)	1.3	2.0	0.16
TIMI minor or major (%)	3.7	3.6	0.81
Planned (staged) PCI within 1 year (%)	13.5	6.3	<0.01
**Efficacy endpoints at 1 year**			
Stent thrombosis (%)	3.3	1.4	<0.01
Myocardial reinfarctions (%)	8.3	3.8	<0.01
Target lesion revascularizations (%)	3.4	1.0	0.038
Combined efficacy endpoint (ST + AMI + TLR) (%)	12.2	4.2	<0.01
**All-cause mortality**			
1 year (%)	17.8	18.7	0.63
5 years (%)	33.8	23.1	<0.01
**Safety endpoints at 1 year**			
Bleeding events after hospital discharge (%)	1.2	3.7	<0.01
Cumulative bleeding events (%)	4.8	6.9	0.07

**Table 4:** Events rates in diabetics vs. non-diabetics stratified by stent-type.

Bare-Metal Stents (BMS)	Diabetics (n = 675)	Non Diabetics (n = 3210)	*p* Value
**Efficacy endpoints at 1 year**			
Stent thrombosis (%)	3.3	1.8	0.016
Myocardial reinfarction (%)	8.3	4.8	<0.01
Target lesion revascularization (%)	3.4	2.6	0.31
Combined efficacy endpoint (ST + AMI + TLR) (%)	12.2	7.5	<0.01
**Safety endpoints at 1 year**			
Bleeding events after hospital discharge (%)	1.2	1.9	0.5
Cumulative bleeding event (%)	4.8	4.3	0.58
**All-cause mortality**			
1 year (%)	17.8	11.7	<0.01
5 years (%)	33.8	19.4	<0.01
**Drug-Eluting Stents (DES)**	**Diabetics** **(n = 879)**	**Non Diabetics** **(n = 3592)**	***p*** **Value**
**Efficacy endpoints at 1 year**			
Stent thrombosis (%)	1.3	1.3	0.9
Myocardial reinfarction (%)	3.8	3.1	0.33
Target lesion revascularization (%)	1.0	1.5	0.26
Combined efficacy endpoint (ST + AMI + TLR) (%)	4.2	3.7	0.49
**Safety endpoints at 1 year**			
Bleeding events after hospital discharge (%)	3.8	3.9	0.61
Cumulative bleeding events (%)	6.9	6.0	0.56
**All-cause mortality**			
1 year (%)	18.7	12.3	<0.01
5 years (%)	23.1	18.6	0.11

## Data Availability

The data presented in this study are available upon reasonable request from the corresponding author. The data are not publicly available due to the sensitive nature of the patients’ data.

## Abbreviations

ACE	angiotensin-converting enzyme
AMI	acute myocardial infarction
ARB	angiotensin II receptor blocker
BMS	bare-metal stent
BSR	Bremen STEMI registry
CABG	coronary artery bypass graft
CAD	coronary artery disease
CI	confidence interval
CK	creatine kinase
DAPT	dual antiplatelet therapy
DES	drug-eluting stent
DC	diabetic cardiomyopathy
ITDM	insulin-treated diabetes mellitus
LBBB	left bundle branch block
MRA	mineralocorticoid receptor antagonist
MVD	multi-vessel disease
OR	odds ratio
PCI	percutaneous coronary intervention
SBP	systolic blood pressure
ST	stent thrombosis
STEMI	ST-elevation myocardial infarction
TIMI	thrombolysis in myocardial infarction
TLR/TVR	target lesion/vessel revascularisation
